# Editorial: Immune studies of SARS-CoV2 and vaccines using preclinical modeling

**DOI:** 10.3389/fimmu.2024.1548624

**Published:** 2025-01-16

**Authors:** Cordelia Dunai, Smita S. Iyer, William J. Murphy

**Affiliations:** ^1^ Department of Dermatology, University of California, Davis, Davis, CA, United States; ^2^ Clinical Infection, Microbiology and Immunology (CIMI), Institute of Infection, Veterinary and Ecological Sciences (IVES), University of Liverpool, Liverpool, United Kingdom; ^3^ Department of Pathology, School of Medicine, University of Pittsburgh, Pittsburgh, PA, United States; ^4^ California National Primate Research Center, University of California, Davis (UC), Davis, CA, United States; ^5^ Department of Internal Medicine, University of California, Davis, Davis, CA, United States

**Keywords:** immunology, virology, SARS-CoV-2, mouse models, preclinical models

## Introduction

Preclinical animal models remain essential for understanding viral pathogenesis as well as for the development and assessment of vaccines and therapeutic strategies. It is extremely important, particularly for immunological studies, to understand species differences and limitations both from the viral pathogenesis as well as immune standpoints (summarized in **Table 1**). Much has been learned about coronaviruses (including pivotal earlier studies on SARS-Co-V) from preclinical animal models which shed insights on both viral pathogenesis as well as immune responses underlying them (reviewed by Collins et al.).

**Table 1:** Pros and cons of preclinical models.

Property of Preclinical Models	Pros	Cons
Mammalian Genetics	High genetic similarity to humans enables relevant insights	Differences in gene expression and immune response can limit applicability
Genetic homogeneity	Studying inbred strains allows replicates, reduces variability, and increases reproducibility	Studying a single strain does not model human genetic diversity
Ease of Genetic Manipulation	Can create transgenic, knockout, or humanized mice to model specific conditions	Human immune components in humanized mice may not function identically
Immune System Studies	Ability to study specific immune pathways in isolation	Mouse immune system differs significantly (e.g. cytokine profiles, T-cell responses) and they are Specific-Pathogen-Free (SPF) and more naïve (or lack diversity in antigen-experienced T and B cell repertoire) than humans
Pathogen Adaptation	SARS-CoV-2 can infect hamsters, ferrets, and non-human primates. SARS-CoV-2 has been adapted to mice for study	Adaptation may alter pathogen and relevance to human infection. Otherwise, K18-hACE2 transgenic mice are needed to study SARS-CoV-2
Vaccination Studies	Useful for preclinical testing of vaccine efficacy	Immune responses to vaccines may not predict human outcomes and the immune response in genetically identically SPF mice does not model human diversity and diverse immune memory responses
Rapid Life Cycle	Short lifespan allows studying disease progression over generations	Short lifespan limits study of chronic or long-term infections. Mostly young animals are studied
Ethics and Accessibility	Considered ethically acceptable with regulations; cost-effective	Ethical concerns still exist, especially with non-human primates
Cost and Maintenance	Low cost and simple housing requirements	Environment and social conditions not reflective of humans. Non-human primate studies are expensive
Drug Testing	Enables high-throughput testing of vaccines and therapeutics	Differences in drug metabolism and immune interactions can lead to poor translatability

It is difficult to estimate how many lives the vaccines saved globally during the COVID-19 pandemic, but approximately 1.5 million lives were saved in the WHO European Region by vaccines which were first developed and tested in preclinical models (1). SARS-CoV-2 virus pathology is complex and systemic, leading to challenges in how best to model it preclinically in different species. A lot of research has focused on the neutralizing antibodies in the immune response against SARS-CoV-2 and their apparent rapid diminution. Previous research has demonstrated aberrant inflammatory responses, particularly in the elderly and obese due to cytokine release syndrome [reviewed in Chegni et al. (2)]. Additionally, it was shown that inflammation can occur in the brain even during mild COVID-19 infection in non-human primate studies offering an initial insight into how other organs, not just the lungs, are affected (3).

Efforts continue to make preclinical models more similar to the human scenarios by modifying genes and the infection method and incorporating factors such as age, obesity, and multiple infections. This Research Topic on preclinical models of SARS-CoV-2 covers this concept of increased translatability as well as strategies to improve vaccines.

## Improving translatability of preclinical models

One of the limitations of the mouse model is the species difference of ACE2, a receptor that SARS-CoV-2 uses to enter cells. Initial studies using a transgenic mice expressing human ACE2 under the K18 promoter (K18-hACE2) transgenic mouse model enabled infection with SARS-CoV-2, however its broader expression is not representative of the clinical scenario and leads to more severe and wider-spread pathology (4–8). Fine-tuning this model, Liu et al. introduced human ACE2 and TMPRSS2 to replace the orthologous mouse gene loci but remain under control of their respective murine promoters. This model enabled investigation of mild infection (with peak viral load at dpi 4) and longer-term studies which are useful for understanding long-term effects of the virus and immune memory.

A novel low inoculum SARS-CoV-2 infection model also enabled study of mild infection and lacked active viral replication in the brain as is present in severe and neuroinvasive SARS-CoV-2 models (Dunai et al.). This is more representative of the majority of clinical cases because it is very rare to find viral protein in human brain parenchyma even in severe COVID-19 cases (9). Despite a lack of viral replication in the brains of SARS-CoV-2-infected K18-hACE2-tg mice, there were elevated pro-inflammatory cytokines and an increase in microglia reactivity indicating indirect immune activation in the brain during SARS-CoV-2 infection (Dunai et al.).

Intriguingly, quite early in the pandemic, an interesting phenomenon emerged—the BCG vaccine was associated with protection from COVID-19 (10). However, this was not replicated in randomized controlled clinical trials (11, 12). Due to the prevalence and health impact of tuberculosis, co-infection is an important issue. Williams et al. investigated co-infection of mice with *M. tuberculosis* and SARS-CoV-2. Counter-intuitively, mice with co-infection actually had a reduced mortality rate. The authors found that the protection was associated with high levels of cytokines including TNF and IFN-γ *in vivo* and *in vitro* with human PBMCs and epithelial cells suggesting that activated innate pathways can lead to broad protective antiviral effects (Williams et al.).

## Strategies to improve SARS-CoV-2 vaccines

It is important to increase vaccine efficiency and optimize delivery, timing, targets, and adjuvants especially since periodic immunizations are now recommended due to persistent generation of new viral variants. While the emphasis in the literature has been on the generation of neutralizing antibodies, it has been shown that generating a strong T cell response is important for long-term immunity although the precise roles of T cells remain poorly understood and understudied (13, 14). Therefore, using multiple B and T cell epitopes for a vaccine is a promising strategy investigated in this Research Topic by Prakash et al. Other promising strategies investigated include: intranasal delivery i.e. a needle-free method with all the advantages of stimulating mucosal immunity by Zhou et al.; coupling vaccine with cytokine stimulation (CCL20/MIP-3α) by Gordy et al.; and stabilizing the spike trimer for a more robust immune response by Avila-Nieto et al. Song et al. went a step beyond most studies which usually do not look at more than one vaccine administration or more than one regimen and found that a heterologous boost resulted in more B cell activation and antibody production. These types of studies are particularly important given the paucity of studies assessing effects of repeated vaccinations on different components of immune responses, as well as means to further optimize them.

## Conclusion

It is important to note that preclinical models have the advantage of controlling for the numerous variables (outbred and diverse populations, sex, age, obesity, prior pathogen exposure, etc) associated with clinical studies (summary of pros and cons in **Table 1**). Added to these are the limitations of clinical studies regarding tissue assessment and assays available, particularly for immune studies. This is the advantage of robust preclinical modelling which offers the ability to control for many of these factors and allow for thorough mechanistic dissection of therapeutic and vaccine strategies as reviewed in this Research Topic (Collins et al.). The emergence of chronic symptoms well after SARS-CoV-2 infection, termed post-acute sequelae of COVID-19 (PASC), also highlight the importance of using preclinical models in long-term studies to determine immunological mechanisms involved. The marked immune changes that arise with normal aging further necessitate more robust preclinical modelling in this area given the populations most at risk for poor outcomes following viral infection (15). Given the tremendous heterogeneity of the human population along with the need for multiple vaccinations due to continuous SARS-CoV-2 exposure, consideration of these variables needs to be undertaken in preclinical modelling to determine effects of different vaccines and vaccine strategies that result in greatest efficacy with minimal adverse effects over time.
